# Transcriptomic Profiling of Embryogenic and Non-Embryogenic Callus Provides New Insight into the Nature of Recalcitrance in Cannabis

**DOI:** 10.3390/ijms241914625

**Published:** 2023-09-27

**Authors:** Mohsen Hesami, Marco Pepe, Maxime de Ronne, Mohsen Yoosefzadeh-Najafabadi, Kristian Adamek, Davoud Torkamaneh, Andrew Maxwell Phineas Jones

**Affiliations:** 1 Department of Plant Agriculture, University of Guelph, Guelph, ON N1G 2W1, Canada; mhesami@uoguelph.ca (M.H.);; 2 Département de Phytologie, Université Laval, Quebec, QC G1V 0A6, Canada; 3 Institut de Biologie Intégrative et des Systèmes (IBIS), Université Laval, Quebec, QC G1V 0A6, Canada; 4 Centre de Recherche et d’innovation sur les Végétaux (CRIV), Université Laval, Quebec, QC G1V 0A6, Canada; 5 Institut Intelligence et Données (IID), Université Laval, Quebec, QC G1V 0A6, Canada

**Keywords:** *Cannabis sativa*, embryonic callus, gene regulation, non-embryogenic callus, plant growth regulators, rooty callus

## Abstract

Differential gene expression profiles of various cannabis calli including non-embryogenic and embryogenic (i.e., rooty and embryonic callus) were examined in this study to enhance our understanding of callus development in cannabis and facilitate the development of improved strategies for plant regeneration and biotechnological applications in this economically valuable crop. A total of 6118 genes displayed significant differential expression, with 1850 genes downregulated and 1873 genes upregulated in embryogenic callus compared to non-embryogenic callus. Notably, 196 phytohormone-related genes exhibited distinctly different expression patterns in the calli types, highlighting the crucial role of plant growth regulator (PGRs) signaling in callus development. Furthermore, 42 classes of transcription factors demonstrated differential expressions among the callus types, suggesting their involvement in the regulation of callus development. The evaluation of epigenetic-related genes revealed the differential expression of 247 genes in all callus types. Notably, histone deacetylases, chromatin remodeling factors, and EMBRYONIC FLOWER 2 emerged as key epigenetic-related genes, displaying upregulation in embryogenic calli compared to non-embryogenic calli. Their upregulation correlated with the repression of embryogenesis-related genes, including LEC2, AGL15, and BBM, presumably inhibiting the transition from embryogenic callus to somatic embryogenesis. These findings underscore the significance of epigenetic regulation in determining the developmental fate of cannabis callus. Generally, our results provide comprehensive insights into gene expression dynamics and molecular mechanisms underlying the development of diverse cannabis calli. The observed repression of auxin-dependent pathway-related genes may contribute to the recalcitrant nature of cannabis, shedding light on the challenges associated with efficient cannabis tissue culture and regeneration protocols.

## 1. Introduction

Plant cells exhibit high plasticity, lending itself to cell differentiation [1]. In response to stresses such as pathogen infection and/or wounding, plants generate tumor-like tissue referred to as calli, which are unorganized cell masses [2]. It has been known for centuries that plants produce callus, the formation of which is a critical part of the grafting process. Callus formation in response to debarking was first documented more than two centuries ago [3]. The etymology of the term “callus” can be traced back to the Latin word “*callum*”, meaning hardness [4]. In the early periods of plant biology, the term “callus” was used to describe the extensive proliferation of cells and the accumulation of “callose” that formed from tissue injury [4]. In 1902, the German botanist Gottlieb Haberlandt observed the formation of undifferentiated masses of cells in the wounds of plant tissue and hypothesized that these callus tissues could be used to regenerate entire plants. In the years that followed, many researchers attempted to induce callus formation in plant tissues using various methods, such as wounding, hormone treatments, and culturing plants on nutrient media [2]. In 1934, the American plant physiologist Frederick White was the first to successfully induce callus formation using aseptic techniques [5], representing a tremendous breakthrough for plant tissue culture. He demonstrated that callus could be produced from tobacco explants cultured on a nutrient agar medium supplemented with coconut milk [5].

The discovery of callus formation through plant tissue culture is a significant milestone in plant biology research [6], sparking the development of many tissue culture techniques and revolutionizing the way that plants are propagated, studied, and manipulated [7]. Among the many applications of plant tissue culture, the production of secondary metabolites with important pharmaceutical, agricultural, and industrial values (e.g., alkaloids, flavonoids, and terpenoids) is of principal interest [8]. It is possible to control the large-scale production of secondary metabolites using in vitro callus cultures. Callus culture has also been used to study plant development and differentiation [2]. By manipulating culture conditions, researchers can induce calli to differentiate into specific cell types, such as roots, shoots, somatic embryos, or even tracheary elements [9]. This has enhanced the study of molecular mechanisms regulating plant development, leading to the discovery of key genes and pathways involved in plant differentiation, and has ultimately been regarded as the foundational approach for modern plant biotechnology [1].

Plant growth regulators (PGRs) play critical roles in regulating callus formation [4,10]. Auxins and cytokinins are the most commonly used hormones in plant tissue culture due to their roles in cell division and differentiation [6]. The balance between these hormones is critical for the induction and maintenance of callus [11]. In general, intermediate ratios of cytokinin and auxin lead to callus formation, while high cytokinin-to-auxin and auxin-to-cytokinin ratios, respectively, result in shoot and root generation [7,12]. The process of callus formation involves a series of molecular events that are still poorly understood. However, researchers have made significant strides in unraveling the underlying mechanisms by generating gain- and loss-of-function callus mutants in model plants [2,9,13]. Despite these advancements, recalcitrant plants of economic importance, such as cannabis, remain understudied and represent considerable research opportunities. 

Callogenesis represents a significant biotechnological approach in cannabis for various purposes, including plant propagation via indirect organogenesis and somatic embryogenesis [14], genetic transformation and genome editing [15], secondary metabolite production [8], and bioenergy generation [16]. Despite recent studies that focus on optimizing in vitro cannabis propagation through callus, regeneration rates have been insufficient, posing significant obstacles for stable gene transformation and genome editing [14,17]. Consequently, a deeper understanding of cannabis callogenesis is necessary and can be attained by examining the transcriptomic profiles of various callus types.

**Figure 1:**
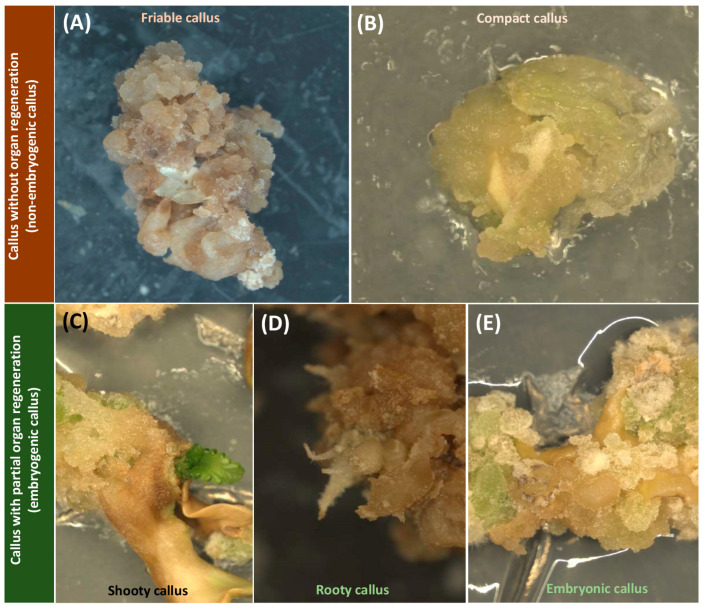
Different types of calli in cannabis including non-embryogenic calli such as (**A**) friable callus and (**B**) compact callus and embryogenic calli such as (**C**) shooty callus, (**D**) rooty callus, and (**E**) embryonic callus.

Callus cells, derived from differentiated somatic cells, exhibit a striking characteristic known as pluripotency, which enables them to generate a diverse range of cell types and organs [1]. However, while callus cells are theoretically capable of differentiating into various cell types, in practice, not all calli are competent to do so. The common notion that callus is a mass of undifferentiated cells is not completely accurate, since there are different types of callus, suggesting that they are not necessarily fully de-differentiated, nor homogenous [6]. It has been shown that the calli of cannabis, like those of other plants, can be categorized into two main groups (i.e., embryogenic and non-embryogenic callus) based on their morphological characteristics (Figure 1). The non-embryogenic callus can be friable (Figure 1A) or compact callus (Figure 1B) with no sign of organ regeneration. While this type of callus is not suitable for regeneration, it can be useful for secondary metabolite production [7]. Embryogenic callus shows apparent organ regeneration such as shoot, root, and somatic embryos, which are respectively known as shooty (Figure 1C), rooty (Figure 1D), and embryonic (Figure 1E) calli [4]. Pluripotency in plant callus cells is characterized by their ability to self-renew, divide, and develop into differentiated cells [9]. Upon callus induction, somatic cells undergo various levels of dedifferentiation, reverting to a more primitive state resembling embryonic cells [6]. This dedifferentiation process is accompanied by significant changes in gene expression profiles, including the downregulation of cell type-specific markers and the upregulation of pluripotency-associated genes [1]. It has also been documented that distinct gene expression profiles can be observed in different types of calli [13].

To better understand gene expression patterns associated with embryogenic and non-embryogenic calli in plants, transcriptomic profiling through RNA sequencing (RNA-Seq) has been performed in different plants such as *Cucumis melo* [18], *Picea balfouriana* [19], *Hordeum vulgare* [20], *Coffea arabica* [21], *Kalopanax septemlobus* [22], and *Gossypium hirsutum* [23]. The mechanisms of gene regulation in the formation of various calli types have been studied, and a plethora of genes with differential expression, including those encoding transcription factors, cell cycle regulators, and secondary metabolism enzymes, have been determined in different plants [2]. For instance, genes encoding somatic embryogenesis receptor-like kinase (SERK), leafy cotyledon1 (LEC1), and auxin response factor (ARF) play pivotal roles in embryonic callus formation [24]. However, until now, there existed no study on global transcriptional changes and their regulation in relation to cannabis callogenesis. As an important industrial and medicinal plant that displays recalcitrance to regeneration, cannabis is a relevant specimen for conducting such a study.

To further analyze the mechanism of formation of different types of calli in cannabis and better guide in vitro culture in the future, this study was performed to compare the transcriptional profiles of various types of calli (i.e., non-embryogenic, rooty, and embryonic callus). Different classes of genes that are upregulated, downregulated, and/or activated in the aforementioned types of cannabis calli are presented and discussed to provide insight into the nature of cannabis recalcitrance.

## 2. Results

### 2.1. Differential Gene Expression and GO Enrichment of Different Types of Calli 

**Figure 2:**
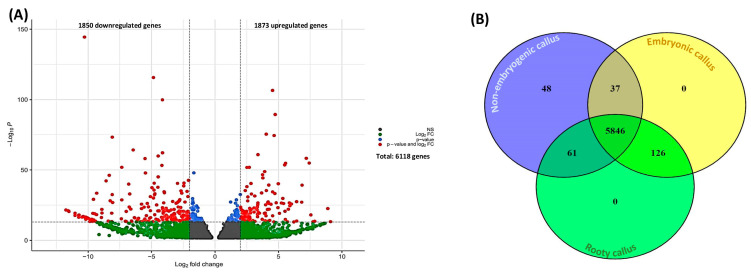
(**A**) Volcano plot and (**B**) Venn diagram of differentially expressed genes.

A high-depth RNA sequencing was achieved with an average of 17.3, 26.3, and 23.6 million 150 bp paired-end reads for non-embryogenic calli, rooty calli, and embryonic calli, respectively. On average, 88.28% of the reads were correctly mapped to the cannabis reference genome. Of 16,417 expressed genes (Table S1), we found that 6118 genes were differentially expressed (1850 downregulated (logFC ≤ −1) and 1873 were upregulated (logFC ≥ 1) genes) within different callus types (Figure 2A and Table S2). Among these genes, 1265 genes were uncharacterized genes (unknown function). 

**Figure 3:**
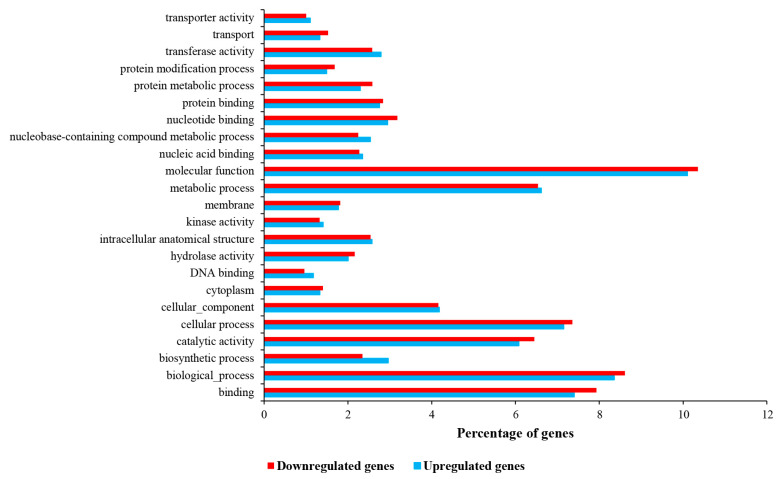
GO enrichment of down- and up-regulated differentially expressed genes.

The GO enrichment analysis revealed that 52.4% of upregulated genes were categorized in the molecular function, followed by the biological process (35.2%) and cellular component (12.4%), with 90, 91, and 90 subcategories, respectively. We then performed hierarchal clustering to classify subcategories based on their frequencies. This resulted in three clusters, where the first cluster covered 45.71% of gene product properties, followed by the second cluster (34.73%) and the third cluster (19.56%). The most pronounced enriched categories were the molecular function (10.11%), biological process (8.36%), binding (7.40%), cellular process (7.15%), metabolic process (6.61%), and catalytic activity (6.08%), respectively (Figure 3). 

The top 10 upregulated genes in embryogenic calli (i.e., embryonic callus and rooty callus) were beta-galactosidase (LOC115706125), receptor-like protein 35 (LOC115725246), programmed cell death protein 4-like (LOC115707713), 1-aminocyclopropane-1-carboxylate oxidase homolog 1 (LOC115723447), guanine nucleotide-binding protein subunit beta-like protein (LOC115709330), 60S ribosomal protein L31 (LOC115705178), F-box/LRR protein (LOC115705507), acid phosphatase 1 (LOC115705442), protein DETOXIFICATION 29 (LOC115714995), and 60S acidic ribosomal protein P1 (LOC115705054) (Table 1). 

On the other hand, GO analysis revealed that 54.66% of downregulated genes were categorized in the molecular function, followed by the biological process (33.52%) and cellular component (11.82%), with 94, 94, and 91 subcategories, respectively. The subcategories were arranged into three clusters using hierarchal clustering. The first cluster covered 47.24% of gene product properties, followed by the second cluster (34.66%) and the third cluster (18.10%). The most pronounced enriched categories were the molecular function (10.35%), biological process (8.61%), binding (7.93%), cellular process (7.36%), metabolic process (6.54%), and catalytic activity (6.45%), respectively (Figure 3). 

The top 10 downregulated genes in embryogenic calli were beta-galactosidase-like (LOC115705878), LRR receptor-like serine/threonine-protein kinase IOS1 (LOC115704132), metal-nicotianamine transporter YSL3 (LOC115716639), guanine nucleotide-binding protein subunit beta-like protein (LOC115709323), protein TIFY 10A-like (LOC115706817), SNF1-related protein kinase regulatory subunit gamma-1 (LOC115711284), peroxidase 24 (LOC115721742), probable 2-oxoglutarate-dependent dioxygenase (LOC115702721), ferredoxin-nitrite reductase (LOC115702931), and neutral ceramidase 2 (LOC115697518) (Table 1).

Among DEGs, 5846 genes were differentially expressed in all types of calli (Figure 2B). In addition, 48 genes were unique to non-embryogenic callus, whereas 126 genes were uniquely expressed in embryogenic calli (i.e., rooty callus and embryonic callus) (Figure 2B and Table S3). The shared gene expression profiles between non-embryogenic callus and embryonic callus were found to consist of 37 genes (Figure 2B and Table S3). In contrast, a larger set of 61 genes was found to be shared between non-embryogenic callus and rooty callus (Figure 2B and Table S3). 

**Figure 4:**
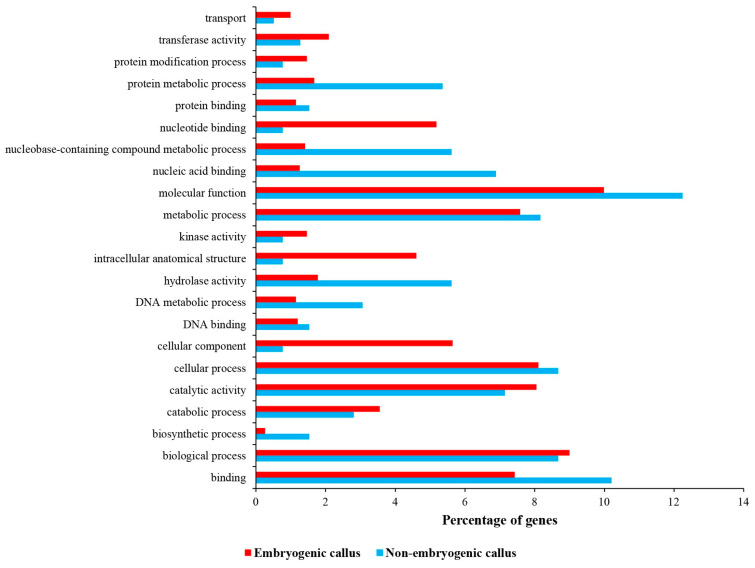
GO enrichment of differentially expressed genes between embryogenic and non-embryogenic calli.

Among the unique genes in non-embryogenic callus, 28 genes were uncharacterized genes (unknown function). The expression magnitudes of unique non-embryogenic callus genes ranged from −11.02 (logFC) to −7.22 (logFC) (Table S3). Cytochrome P450 71D9 (LOC115695663), cytidine deaminase 1 (LOC115705713), CASP-like protein 4D1 (LOC115707613), pentatricopeptide repeat-containing protein (LOC115710618), F-box/LRR-repeat protein (LOC115707558), mitogen-activated protein kinase 6 (LOC115704245), and probable S-adenosylmethionine-dependent methyltransferase (LOC115710298) are some examples of unique genes in non-embryogenic callus (Table S3). Based on the results of the GO, 65.05% of product properties of unique genes in non-embryogenic callus were categorized in the molecular function, followed by the biological process (32.14%) and cellular component (2.81%), respectively. In addition, the molecular function contained 28 subcategories, the biological process contained 27 subcategories, and the cellular component contained 11 subcategories. The most pronounced enriched categories were the molecular function (12.25%), binding (10.2%), biological process (8.67%), cellular process (8.67%), metabolic process (8.16%), and catalytic activity (7.14%), respectively (Figure 4). The lowest pronounced enriched category was the generation of precursor metabolites and energy (0.26%). 

Among the unique genes in embryogenic calli (i.e., rooty callus and embryonic callus), 58 genes were uncharacterized genes (unknown function). The expression magnitudes of unique non-embryogenic callus genes ranged from 3.97 (logFC) to 9.1 (logFC) (Table S3). The mediator of RNA polymerase II transcription subunit 15a (LOC115708122), B3 domain-containing transcription factor VRN1 (LOC115697055), transcription factor MYB60 (LOC115721852), ATP-dependent DNA helicase pfh1-like (LOC115700347), ethylene-responsive transcription factor ERN1-like (LOC115723957), NAC domain-containing protein 43-like (LOC115708111), and G2/mitotic-specific cyclin S13-7 (LOC115718744) are some examples of unique genes in embryogenic callus (Table S3). Based on the results of the GO, 57.69% of the product properties of unique genes in embryogenic callus were categorized in the molecular function, followed by the biological process (31.80%) and cellular component (10.51%), respectively. In addition, the molecular function contained 37 subcategories, the biological process contained 37 subcategories, and the cellular component contained 28 subcategories. The most pronounced enriched categories were the molecular function (10%), biological process (9%), cellular process (8.11%), catalytic activity (8.05%), metabolic process (7.58%), and binding (7.43%), respectively (Figure 4). The lowest pronounced enriched category was anatomical structure development (0.11%). It is notable that some genes unique to embryogenic callus were categorized in categories not found in non-embryogenic callus (i.e., response to stress, cellular component organization, carbohydrate binding, carbohydrate metabolic process, cell wall, cytoplasm, DNA-binding transcription factor activity, external encapsulating structure, extracellular region, membrane, nuclease activity, nucleus, transcription regulator activity, and transporter activity).

### 2.2. Differentially Expressed Phytohormones-Related Genes 

**Figure 5:**
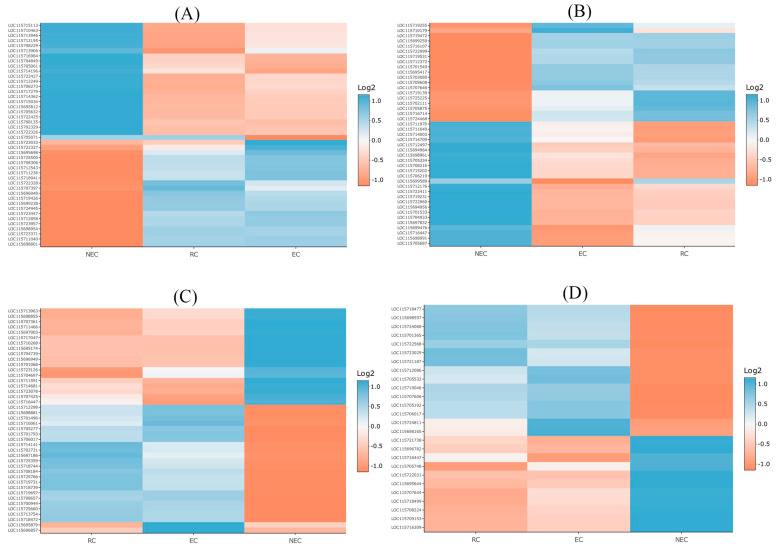
Differentially expressed phytohormones-related genes including (**A**) ethylene, (**B**) auxin, (**C**) cytokinin, and (**D**) abscisic acid. (See Table S4 for gene descriptions and more details). EC: embryonic callus, RC: rooty callus, NEC: non-embryogenic callus.

**Figure 6:**
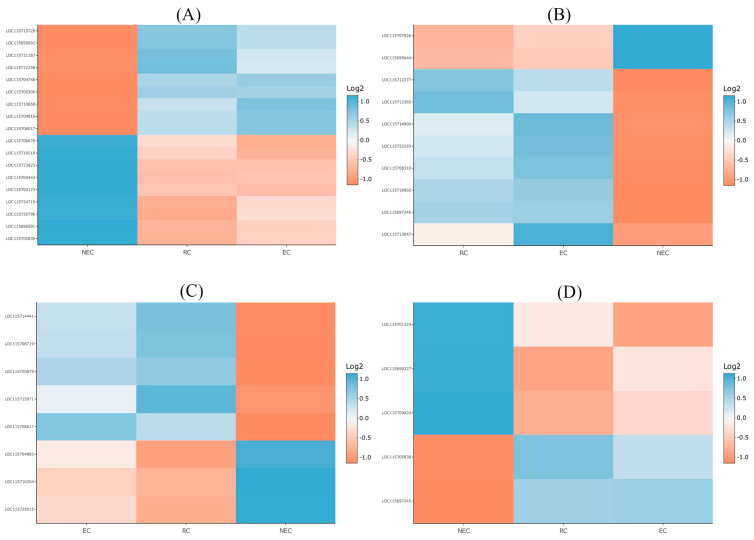
Differentially expressed phytohormones-related genes including (**A**) gibberellic acid, (**B**) salicylic acid, (**C**) brassinosteroid, and (**D**) jasmonic acid. (See Table S4 for gene descriptions and more details). EC: embryonic callus, RC: rooty callus, NEC: non-embryogenic callus.

Genes related to the biosynthesis and signaling of phytohormones play pivotal roles in the formation of different types of calli. In total, 196 phytohormone-related genes were differentially expressed in different types of calli (Table S4). Of these, 65 phytohormone-related genes were downregulated (logFC ≤ −1) in embryogenic calli (i.e., rooty callus and embryonic callus), while 64 were upregulated (logFC ≥ 1) (Table S4). Ethylene-related genes were the phytohormone class with the most abundant DEGs (44 genes, Figure 5A), followed by auxin (43 genes, Figure 5B), cytokinin (42 genes, Figure 5C), abscisic acid (26 genes, Figure 5D), gibberellic acid (18 genes, Figure 6A), salicylic acid (10 genes, Figure 6B), brassinosteroid (8 genes, Figure 6C), and jasmonic acid (5 genes, Figure 6D), respectively. 

### 2.3. Transcription Factor-Related Genes

Transcription factors play crucial roles in regulating the complex processes of callus development. Our results showed that 42 different transcription factor classes were differentially expressed in different types of calli (Table S5). MYB was the first class of transcription factors with the most abundant genes (45 genes), followed by C2H2 (40 genes), ERF (26 genes), AP2 (24 genes), bHLH (21 genes), bZIP (21 genes), B3 (20 genes), WRKY (19 genes), GRAS (16 genes), NAC (16 genes), ARF (15 genes), G2-like (12 genes), ARR-B (11 genes), C3H (11 genes), GATA (10 genes), FAR1 (9 genes), HD-ZIP (9 genes), MADS (9 genes), HB-other (8 genes), LBD (8 genes), Trihelix (8 genes), CO-like (6 genes), Dof (6 genes), TALE (6 genes), TCP (6 genes), BES1 (5 genes), CAMTA (5 genes), HSF (5 genes), NF-YB (5 genes), SBP (5 genes), NF-YA (4 genes), DBB (3 genes), RAV (3 genes), GRF (2 genes), NF-YC (2 genes), YABBY (2 genes), ZF-HD (2 genes), WOX (1 gene), EIL (1 gene), SRS (1 gene), and Whirly (1 gene), respectively (Table S5).

Specific genes with known functions in the formation of different types of calli were individually searched in the DEGs. For instance, ARF 19 (LOC115709608), AINTEGUMENTA-like 5 (ALI5, LOC115695698), APETALA2/ethylene response factor (AP2/ERF, LOC115725443), and cyclin-D3-3 (CYCD3-3, LOC115702731), which are involved in callus formation, were differentially expressed in all types of calli. On the other hand, many genes with crucial roles in somatic embryogenesis (e.g., LEAFY COTYLEDON 2 (LEC2), BABY BOOM (BBM, LOC115718863), and AGAMOUS-LIKE15 (AGL15, LOC115710309)) were not differentially expressed, which might be due to the epigenetic regulations. 

### 2.4. Epigenetic Machinery-Related Genes 

The reason for the lack of expression of embryogenesis-related genes can open a new window in our understanding of the recalcitrant nature of cannabis to in vitro culture. Hence, DEGs were searched to detect epigenetic machinery-related genes. In general, 247 DEGs related to different epigenetic machineries (e.g., chromatin remodeling, DNA methylation, and histone modification) were detected (Table S6). Among these genes, 61 genes were downregulated (logFC ≤ −1) in embryogenic calli, while 68 genes were upregulated (logFC ≥ 1) in embryogenic calli (Table S6). For instance, histone deacetylase 6 (HDAC6, LOC115703044) and CHROMATIN REMODELING 35 (LOC115724492), which target embryogenesis-related transcription factors (e.g., LEC1 and LEC2), were upregulated in embryogenic callus (Table S6), showing the pivotal roles played by epigenetics in cannabis somatic embryogenesis.

## 3. Discussion

The study of different types of calli is of utmost importance in the field of plant biotechnology and to a deeper understanding of the fundamental factors leading to plant regeneration and/or recalcitrance [25,26,27]. Callus is a mass of undifferentiated cells that can theoretically be induced to differentiate into different plant organs, such as roots, shoots, and embryos [4,7]. The ability to produce callus is essential for various plant tissue culture applications, including genetic transformation [15], micropropagation [28], secondary metabolite production [8], and bioenergy generation [16]. While cannabis readily forms callus, most researchers have been unable to efficiently regenerate plants from it [14,15,29,30,31]. New information related to the genes involved in callus formation and subsequent development will aid in better understanding the cause of recalcitrance in cannabis and help to develop strategies for overcoming this recalcitrant nature.

In the current study, different types of cannabis calli (i.e., non-embryogenic, rooty, and embryonic callus) were produced in a medium containing auxin (i.e., 2,4-D) and cytokinin (i.e., kinetin), which has been shown to produce a variety of callus types. These callus types include both non-embryogenic and embryogenic calli. Embryogenic calli can result in the formation of different organs, including shoots, roots, or somatic embryos. These callus types were used to study differential gene expression profiles to gain insight into the nature of recalcitrance and regeneration. It is well-documented that auxin and cytokinin play fundamental roles in callogenesis [32,33]. Previous studies in model plants have also revealed that callogenesis using auxin and cytokinin is controlled through complex gene regulatory networks [2,4,6]. 

Recent genetic data from *Arabidopsis* have greatly enhanced our comprehension of the molecular mechanisms that control embryogenic callus formation [6]. Specifically, studies related to embryonic callus formation have elucidated the delicate equilibrium between cell proliferation in the apical domain, the processes of programmed cell death (PCD), and terminal differentiation in the basal domain [24,34,35]. In line with genetic data from model species, our results showed that the programmed cell death protein 4-like gene (LOC115707713) was upregulated in embryogenic calli.

Embryogenic callus forms in response to exogenous PGRs in tissue culture media and is initiated from a group of cells (or even a single cell) with similar genetic backgrounds and morphologies [36]. These stimuli act through receptor-like proteins (RLPs) to regulate several cellular mechanisms and ultimately alter gene transcription patterns [37]. Indeed, RLPs regulate several cellular mechanisms to support cell differentiation and/or redifferentiation as well as adaptation under in vitro culture conditions [38]. Our results also showed that RLP35 (LOC115725246) was upregulated in embryogenic callus (i.e., rooty callus and embryonic callus), showing the critical roles of RLPs in embryogenic callus formation.

Embryogenic callus formation is accompanied by changes in the cellular components and structure of the cell wall [24,39]. The GO analysis of our study indicated that 12.4% of upregulated gene product properties were categorized in the cellular components category. Modifications of the cell wall are crucial for maintaining the balance of forces required for the cellular architecture, the cell shape, and the determination of the division plane [40]. In addition, cell walls play pivotal roles in connecting cells with their neighboring cells. A wide range of signaling factors transported to cell walls from the cytoplasm can be exported through the apoplast into neighboring cells, which promotes embryogenic callus formation [41,42,43]. In line with previous studies in other plant species, our result also showed that the most pronounced enriched GO categories for upregulated genes were the molecular function, biological process, binding, cellular process, metabolic process, and catalytic activity, respectively. The high representation of molecular function among the enriched categories suggests the importance of altered molecular functions in the regulation of embryogenic potential. Changes in molecular functions can directly influence cellular processes and signaling events to ultimately impact developmental processes associated with embryogenesis [39]. The specific molecular functions enriched in the upregulated genes must still be elucidated, but they likely play essential roles in modulating cellular processes required for embryogenic potential [2,24,40,44].

Several cell wall-modifying enzymes (e.g., β-galactosidase and 1,4-alpha-glucan-branching enzyme) are typically differentially expressed in embryogenic calli [6,24]. Based on our results, embryogenic callus formation is accompanied by the up-regulation of β-galactosidase (LOC115706125), which removes terminal galactosyl residues from galactolipids, glycoproteins, and carbohydrates and can thus modify the architecture and structure of the cell wall, as well as other properties, such as intercellular attachments [45]. Similar results were previously reported in plants such as *Pinus radiata* [46], cassava [47], and cotton [23]. Our results also showed the upregulation of 1,4-alpha-glucan-branching enzyme 2-2, chloroplastic/amyloplastic-like gene (LOC115725315) in embryogenic callus. This gene plays a key role in the starch synthesis pathway through the removal of 1, 4-alpha-linked oligosaccharides and, subsequently, the formation of the alpha-1, 6-glucosidic linkages in glycogen [48]. The 1, 4-alpha-glucan branching enzyme can ultimately alter the mechanical properties of the cell wall [48]. Geraniol 8-hydroxylase is another gene involved in cellular components by producing indole-3-acetaldoxime from tryptophan, resulting in the promotion of embryogenic callus formation [49]. Our results showed that this gene (LOC115725467) was upregulated in the embryogenic callus. The 60S ribosomal proteins (e.g., 60S ribosomal protein L31, 60S acidic ribosomal protein P1) play important roles related to embryogenic callus formation due to their functions in protein production [37,40,50]. Our results showed that these proteins (LOC115705178 and LOC115705054) were upregulated in embryogenic calli.

Importantly, the upregulation of cell wall-related signaling transduction genes such as those for guanine nucleotide-binding proteins is necessary for embryogenic callus formation [51,52]. Our results also showed that guanine nucleotide-binding protein subunit beta-like protein (LOC115723447) was upregulated in embryogenic callus. Similar to our results, Polesi et al. [51] reported that guanine nucleotide-binding protein subunit beta-like protein is a unique protein in the embryogenic callus of *Guadua chacoensis*. The serine carboxypeptidase gene plays an important role in secondary metabolite biosynthesis [53]. The expression of this gene as the signal transduction gene for embryogenic callus in citrus [53] and Douglas fir [54] indicates its crucial role in embryogenic callus formation. In line with these studies, our results revealed that serine carboxypeptidase (LOC115700502) was upregulated in embryogenic calli. The SNF1-related protein kinase regulatory subunit gamma-1-like is connected to another signal transduction cascade that regulates the expression of carbohydrate metabolism-related genes and thereby plays a crucial role in embryogenic callus formation [55]. Our results showed the upregulation of this gene (LOC115711285), demonstrating its role in cell wall-related signaling transduction in embryogenic calli.

Our results also demonstrated that protein DETOXIFICATION 29 (LOC115714995) was upregulated in the embryogenic calli. Since high levels of unstable and damaged proteins are usual in callogenesis due to stressful in vitro conditions, detoxification proteins are crucial for embryogenic callus formation [50].

In addition to top up- and top down-regulated genes, we performed a specific search to find phytohormone-related genes. Genes related to the biosynthesis and signaling of phytohormones such as auxin [56], cytokinin [57], gibberellic acid [58], abscisic acid [59], ethylene [60], salicylic acid [11], jasmonic acid [61], and brassinosteroid [62] play pivotal roles in the formation of different types of calli. Our results showed that 196 phytohormone-related genes were differentially expressed in different types of calli, demonstrating the importance of PGRs in callogenesis.

In addition to PGRs, transcription factors play crucial roles in regulating the complex process of indirect somatic embryogenesis [13]. Through their ability to bind to specific DNA sequences, transcription factors control the expression of genes involved in embryogenic cell formation, proliferation, and differentiation [63]. They act as key molecular switches, orchestrating the activation or repression of target genes, thereby modulating the intricate signaling networks and molecular events required for somatic embryogenesis [13]. By fine-tuning the balance of gene expression, these transcription factors govern the transition of somatic cells into embryogenic cells. Subsequent embryo development can then proceed, making such transcription factors pivotal players in the regulation of this highly efficient and valuable plant regeneration pathway [2]. We observed 42 classes of transcription factors that were differentially expressed in different types of calli. Besides transcription factors, epigenetic modifications significantly impact the formation of different types of calli [9]. Epigenome reprogramming, facilitated by chromatin-modifying factors, is responsible for altering the chromatin states of genes [64]. These factors play critical roles in orchestrating comprehensive changes to the global transcriptome during callogenesis [2,9]. Three distinct mechanisms (i.e., DNA methylation, histone modification, and nucleosome remodeling) are involved in modifying the chromatin structure [65,66]. As a result, the compaction of chromatin is either decreased or increased, leading to alterations in the accessibility of chromatin for the transcription machinery [67]. Our results showed that 247 epigenetic-related genes were differentially expressed in all types of calli. To elucidate the gene regulatory networks of cannabis callogenesis, specific genes with known functions in *Arabidopsis* related to the formation of different types of calli were individually analyzed for expression profiles in non-embryogenic callus and embryogenic callus.

Previous studies using *Arabidopsis* have demonstrated that various members of the ASYMMETRIC LEAVES2-LIKE family, also referred to as LATERAL ORGAN BOUNDARIES DOMAIN (LBD) transcription factors, including LBD16 and LBD29, mediate auxin signaling downstream of AUXIN RESPONSE FACTOR 7 (ARF7) and ARF19 [68,69]. LBD16 activates E2 PROMOTER BINDING FACTOR a (E2Fa), a core cell cycle regulator, leading to enhanced cell proliferation [70]. Additionally, LBD29 plays a role in cell wall modification processes [71]. PECTIN METHYLESTERASE (PME), a cell wall modifier, is one of the targets of LBD29, ultimately promoting callogenesis [72]. Our results revealed that ARF19 (LOC115709608), several LBDs (e.g., LBD4 (LOC115708902), LBD12 (LOC115720935), LBD19 (LOC115706345), LBD30 (LOC115705047), LBD39 (LOC115713617), and LBD40 (LOC115699573)), and PME (LOC115704128) were differentially expressed among certain types of cannabis calli. However, ARF 7, LBD16, LBD 29, and E2Fa were not differentially expressed in our study, suggesting the importance of other pathways in cannabis callogenesis.

The role of the cytokinin-dependent pathway in callogenesis is less clear than that of the auxin-dependent pathway. However, type-B RESPONSE REGULATORs (RRs) can be considered a critical component in the cytokinin-dependent pathway. Previous studies showed that RR 2 overexpression promotes Arabidopsis callogenesis in cytokinin-containing media. A potential target of RR2 in promoting the reentry to the cell cycle is CYCD3;1. In addition, previous studies showed that the expression of CYCD3;1 is upregulated in cytokinin-containing media. In line with these results in *Arabidopsis*, our results showed that RR (LOC115711591) and CYCD3;1 (LOC115718739) were expressed in all types of cannabis calli.

Another candidate, APETALA2/Ethylene-responsive factor (AP2/ERF), plays a pivotal role in cytokinin-mediated callogenesis through linking cytokinin signaling to cell cycle regulation. The direct activation of OBF BINDING PROTEIN1 (OBP1) and CYCD3;1 is mediated by AP2/ERF. The OBP1 gene shortens the G1 phase duration, which facilitates reentry to the cell cycle. In addition, OBP1 can directly activate the CYCD3;3 gene, which ultimately results in callogenesis. Consistent with findings in model plants, our results revealed that AP2/ERF (LOC115725443), OBP1 (LOC115719739), CYCD3;1, and CYCD3;3 (LOC115702731) were differentially expressed in all types of cannabis calli, emphasizing the critical role of the cytokinin-dependent pathway in cannabis callogenesis.

Overall, our findings indicated that all the genes associated with the cytokinin-dependent pathway were differentially expressed. These results are key to unlocking mysteries related to the recalcitrant nature of in vitro cannabis and serve as important information for overcoming such obstacles. It is well-documented that the expression of auxin-related genes in non-recalcitrant plants is significantly higher than that of cytokinin genes. Previous studies in recalcitrant plants showed that if auxin-related genes are upregulated to a higher degree than cytokinin genes, the non-embryogenic callus can be converted to embryogenic callus [1,13,18,19,22,23,73]. Hence, the limited expression levels of auxin-dependent pathway-related genes may account for the recalcitrant nature of cannabis.

**Figure 7:**
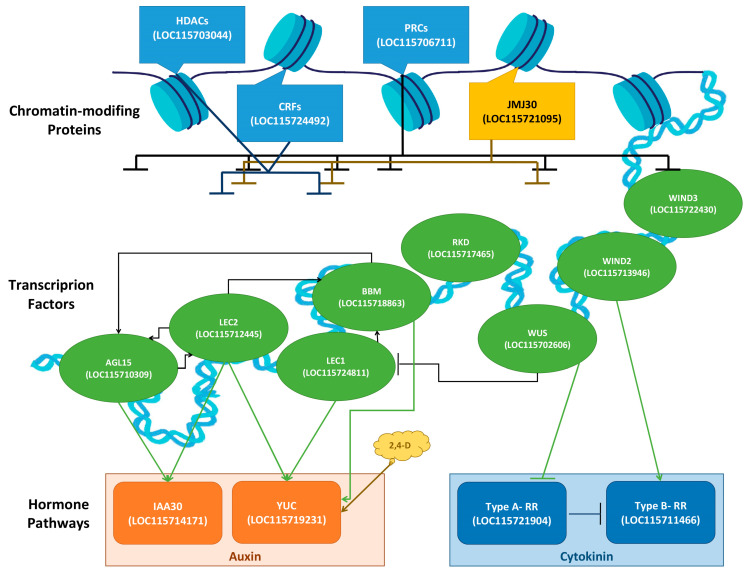
Schematic representation of the molecular regulation of *Cannabis sativa* somatic embryogenesis.

Callus formation appears to exhibit significant heterogeneity, as observed in the formation of calli derived from lateral root primordia, where the expression of root meristem markers is only partially regulated [74]. Notably, callus cells demonstrate the ability to differentiate into rooty calli and embryonic calli, suggesting the existence of shared pathways between these callus types [6]. The establishment and maintenance of pluripotency in plant callus cells are orchestrated by an intricate network of molecular regulators [2]. Transcription factors, such as members of the LEC1, LEC2, and WUSCHEL-related homeobox (WOX) families, play crucial roles in promoting pluripotency by activating key regulatory pathways [13,24]. These transcription factors interact with chromatin modifiers to regulate the epigenetic landscape of callus cells [9] (Figure 7).

The acetylation of histone 3 (H3) and H4, which are tightly regulated by histone deacetylases (HDACs) and histone acetyltransferases (HATs), plays a pivotal positive role in expressing embryogenesis-related genes [9]. Tanaka et al. [75] showed that the upregulation of HDACs repressed embryogenic markers such as ABSCISIC ACID INSENSITIVE 3 (ABI3), FUSCA3 (FUS3), the LEAFY COTYLEDON 1 (LEC1), and LEC2, suggesting that embryogenic calli failed to make the transition necessary to allow for somatic embryogenesis. Our results showed that HDAC (LOC115703044) was upregulated in embryogenic calli. In addition, LEC2 (LOC115712445) was not expressed in all types of calli, suggesting that HDAC upregulation had resulted in the repression of LEC2 (Figure 7).

The chromatin remodeling factors (CRFs) mediate crosstalk between histone acetylation and histone methylation. Previous studies revealed that the upregulation of CRFs leads to the repression of LEC1 and LEC2, resulting in the production of the changes necessary for embryogenic calli to enable somatic embryogenesis [44,76]. Indeed, CRFs may function by guiding both H3 lysine 27 trimethylation (H3K27me3) and HDACs [9]. Our results showed that CRFs such as CHROMATIN REMODELING 35 (LOC115724492) were upregulated in embryogenic calli. Thus, it seems that the combination of HDAC and CRF ensures the silencing of LEC2 in cannabis calli and likely inhibits somatic embryogenesis from occurring in embryogenic calli (Figure 7).

Critical roles related to histone modification are played by JUMONJI 30 (JMJ30) [65], the upregulation of which can repress WUS, LEC1, and LEC2 [77]. Our results showed that JMJ30 (LOC115721095) was downregulated in embryogenic calli. However, a previous study demonstrated that either Polycomb repressive complex (PRCs) or JMJ30 activity can be sufficient to suppress somatic embryogenesis [77,78].

It has been shown that members of the PRCs such as EMBRYONIC FLOWER 2 (EMF2), SWINGER (SWN), and CURLY LEAF (CLF) play crucial roles related to indirect somatic embryogenesis [13]. Chanvivattana et al. [79] revealed that double loss-of-function mutants in PRCs such as EMF2 in *A. thaliana* led to ectopic root formation and indirect somatic embryogenesis. Bouyer et al. [80] showed that PRCs repress embryogenesis-related genes such as LEC1, LEC2, WOUND INDUCED DEDIFFERENTIATION 3 (WIND3), WUSCHEL (WUS), BABY BOOM (BBM), and AGAMOUS-LIKE15 (AGL15). Ikeuchi et al. [81] also showed that PRC mutants overexpressing WIND3 and LEC2 promoted indirect somatic embryogenesis. In contrast to many embryogenesis-related genes, the expression of LEC1 depends on the type of explant and the presence of PGRs such as 2,4-D in the medium [82]. Our results also showed that LEC1 (LOC115724811) was upregulated in embryogenic calli, which might be due to the presence of 2,4-D in the medium. Mozgová et al. [82] showed that PRCs suppress somatic embryogenesis through the repression of their targets such as LEC2, AGL15, and BBM. However, PRCs had no effect on LEC1 expression in the presence of 2,4-D [82]. In line with these studies, our results showed that EMF2 (LOC115706711) was differentially expressed in all types of calli, which resulted in the repression of WIND3 (LOC115722430), BBM (LOC115718863), LEC2, and AGL15 (LOC115710309) (Figure 7). One of the members of the AINTEGUMENTA-LIKE (AIL) group of APETALA2/ETHYLENE RESPONSE FACTOR (AP2/ERF), BBM, plays an important role in somatic embryo induction [1,2]. The expression of BBM and AIL/PLETHORA (PLT) genes (e.g., PLT1, PLT2, PLT3, PLT5, or PLT7) induces somatic embryogenesis, resulting in the formation of somatic embryos [13]. It has also been shown that AIL5 overexpression induces somatic embryogenesis [83]. Generally, the overexpression of all members of AIL proteins, except AINTEGUMENTA (ANT) and AIL1, leads to the induction of somatic embryogenesis [13]. However, in a study, Zhang et al. [14] explored the utility of various morphogenic genes, such as SHOOT MERISTEMLESS (STM), ISOPENTENYL TRANSFERASE (IPT), GROWTH-REGULATING FACTOR (GRF), GRF-INTERACTING FACTOR (GIF), BBM, and WUS, in enhancing the process of indirect shoot regeneration in hemp cultivars. However, the introduction of these morphogenic genes only resulted in a marginal increase of less than 6% regeneration frequency. It can be concluded that the epigenetic state of callus cells might suppress the activity of these transcription factors, thus contributing to the recalcitrance of cannabis towards indirect regeneration.

Another transcription factor that plays an important role in the induction of somatic embryogenesis is RWP-RK DOMAIN-CONTAINING 4 (RKD4)/GROUNDED (GRD) [1]. While RKD4 is the only member of the RKD family that induces somatic embryogenesis, other members of RKD influence the development of the embryo sac [13]. However, no expression of RKD (LOC115717465) was detected in our study.

Previous studies showed that LEC1, LEC2, FUS3, and AGL15 regulate auxin production and signaling for the induction of somatic embryogenesis, leading to the production of somatic embryos [2]. The YUCCA10 (YUC10) gene encoding an auxin synthesis enzyme is induced by LEC1 [84]. In line with previous studies in model plants, our results also showed that YUC10 (LOC115719231) was differentially expressed in all types of calli. GYUC2 and YUC4 genes are activated by LEC2 [85]. To modulate the auxin-mediated signaling, the expression of INDOLE ACETIC ACID INDUCIBLE 30 (IAA30) as a negative auxin signaling regulator is induced by LEC2 and AGL15 [84]. Our results also showed that YUC2 (LOC115704335), YUC4 (LOC115702268), and IAA30 (LOC115714171) were not expressed due to the absence expression of LEC2 and AGL15.

Previous studies showed that a low ratio of gibberellic acid (GA) to abscisic acid (ABA) can promote somatic embryogenesis [13]. It has also been shown that AGL15 can be a positive regulator for the GIBBERELLIN 2-OXIDASE6 (GA2ox6) gene encoding a GA-degrading enzyme and a negative regulator for the GA3ox2 gene which generally causes a reduction in the endogenous GA concentration [86]. Furthermore, GA biosynthesis is downregulated by FUS3 through activating ABA biosynthesis and repressing GA3ox1 and GA3ox2 [87]. In contrast, our results showed that gibberellin dioxygenase genes (e.g., LOC115704748, LOC115705300, and LOC115710658) were upregulated in embryogenic calli, which is considered to be an additional sign of a failure to induce somatic embryogenesis in embryogenic callus.

Another transcription factor, WUSCHEL (WUS), plays an important role in embryogenic callus formation [4]. The role of WUS in somatic embryogenesis has been previously shown in *Arabidopsis* [88]. Type A-RR is the target of WUS. In fact, WUS represses Type A-RR and promotes embryogenic callus formation [89]. Our results also showed that WUS (LOC115702606) was upregulated in embryogenic calli which resulted in the repression of Type A-RR (LOC115721904), demonstrating the role of this gene in embryogenic callus formation. In addition, the expression of WIND2 by regulating type B-RR as its target promotes embryogenic callus formation in the cytokinin-dependent pathway [90]. Our results also showed that WIND2 (LOC115713946) was differentially expressed, which resulted in the expression of Type B-RR (LOC115711466), and finally promoted embryogenic callus formation. Overall, our findings indicated that the expression levels of genes associated with the cytokinin-dependent pathway resulted in embryogenic callus formation. However, the repression of auxin-dependent pathway genes led to the suppression of somatic embryogenesis. As discussed, for the callogenesis, it seems that the repression of auxin-dependent pathway-related genes may account for the recalcitrant nature of cannabis (Figure 7).

## 4. Materials and Methods

### 4.1. Plant Material Source, Callus Formation, and Sampling

**Figure 8:**
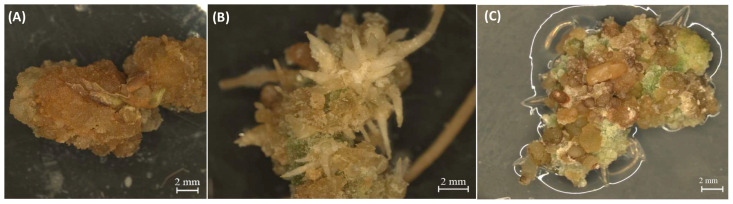
Different types of cannabis calli from this study. (**A**) Non-embryogenic callus, (**B**) rooty callus, and (**C**) embryonic callus are shown.

The current study was carried out on drug-type cannabis (UP305; Up Cannabis, ON). The callogenesis was performed based on our previously developed protocol [17]. Different types of calli including non-embryogenic callus (Figure 8A) and embryogenic callus (i.e., rooty callus (Figure 8B) and embryonic callus (Figure 8C)) were all obtained based on visual cues, including lack of organization and signs of de novo roots and polarization. Leaf explants from in vitro plantlets were cultured on a medium composed of MS [91] basal salts (Phytotechnology Laboratories, Overland Park, KA, USA), B_5_ [92] vitamins (Phytotechnology Laboratories, Overland Park, KA, USA), 0.7% agar (Fisher Scientific, Waltham, MA, USA), 3% sucrose, 0.38 mg/L kinetin (Thermo Fisher Scientific, Waltham, MA, USA), and 0.46 mg/L 2,4-dichlorophenoxyacetic acid (2,4-D, Thermo Fisher Scientific, Waltham, MA, USA). The cultures were kept in a growth chamber at 25 ±  2 °C under a 16 h photoperiod with 40  ± 5 μmol m^−2^ s^−1^ light intensity. In order to minimize the background signals of gene expression that may arise from different culture boxes, non-embryogenic calli, rooty calli, and embryonic calli were selected from 10 individual culture boxes after 90 days. The calli were then combined into a single tube, flash frozen, and stored at −80 °C. This procedure was repeated three times to obtain three biological replicates of each.

### 4.2. RNA Isolation and RNA Sequencing

To isolate RNA samples, pooled calli were placed in 1 mL of TRIzol™ (Thermos Fisher Scientific, Waltham, MA, USA) reagent before being crushed within an RETSCH MM 400 mixer mill (Fisher Scientific, Waltham, MA, USA). Total RNAs were isolated using the TRIzol™ protocol according to the manufacturer’s instructions in conjunction with DNase. The integrity of the total purified RNA was determined using a 2100 Bioanalyzer™ (Agilent Technologies Inc., Santa Clara, CA, USA), and only the three best samples per condition were used for library preparation.

To prepare RNA libraries for sequencing, the quantity of starting material was adjusted to 1 µg per sample. The preparation of mRNA-seq was facilitated with the use of the NEBNext^®^ Ultra™ II directional RNA library Prep kit for Illumina, according to the manufacturer’s instructions, at the Genomic Analysis Platform of the Institut de Biologie Intégrative et des Systèmes (Université Laval, Québec, QC, Canada). The quality of the cDNA library was evaluated using an Agilent 2100 Bioanalyzer™. The RNA libraries were then sequenced on an Illumina NovaSeq 6000 platform (2 × 150 bp read) at the Centre d’expertise et de services Génome Québec (Montreal, QC, Canada).

### 4.3. Transcriptomic Analyses

Raw reads underwent a preprocessing and filtering procedure to ensure good-quality data were obtained. Specifically, Trimmomatic [93] was used to remove Illumina adapters from the reads, followed by the use of the FASTX Toolkit (http://hannonlab.cshl.edu/fastx_toolkit/index.html, accessed on 4 April 2023 version 0.0.13.2) to trim nucleotides with quality scores below 25 and remove reads containing less than 70% base pairs. Clean reads were subsequently aligned to the *Cannabis sativa* reference genome (cs10; https://www.ncbi.nlm.nih.gov/assembly/GCA_900626175.2, accessed on 4 April 2023) using the STAR version 2.7.0a [94]. Gene abundance was inferred by utilizing the cs10 reference genome annotation obtained from NCBI (https://ftp.ncbi.nlm.nih.gov/genomes/all/GCF/900/626/175/GCF_900626175.2_cs10/GCF_900626175.2_cs10_genomic.gff.gz, accessed on 4 April 2023) to generate fragment per kilobase of transcript per million (FPKM) mapped reads values. Afterward, FeatureCounts v. 1.5.0 [95] was used to quantify the number of reads mapped to each detected gene, and differential expression analysis was conducted using the DESeq2 R package [96]. Genes with fewer than 50 reads were removed from the analyses, and genes with an adjusted *p*-value of no more than 0.05 were considered differentially expressed. The fold change for each condition was compared based on the average of different condition sets. The expression values of both the rooty callus and the embryonic callus, referred to as embryogenic callus, were averaged, and these averages were then compared to the average of the non-embryogenic type.

The gene sequences were retrieved using the biomartr R package version 1.0.2 [97] to call the correct gene-associated names, and the data were used to help elucidate the gene ontology (GO) enrichment of differentially expressed genes (DEGs) using rentrez version 1.2.3 and biomaRt version 2.50.3 [98].

The Ortho DB V11 [99] database provided by the National Center for Biotechnology Information (NCBI) was used to identify homologous genes of *Arabidopsis thaliana* in *Cannabis sativa*. We selected a set of *Arabidopsis thaliana* genes of interest for which we wanted to identify homologs in *Cannabis sativa*. These genes were chosen based on their known functions in callogenesis in *Arabidopsis* and their potential relevance to the formation of different types of calli in *Cannabis sativa*.

## 5. Conclusions

This study offers comprehensive insight into the dynamics of gene expression and molecular mechanisms that direct the development of various types of cannabis calli. These findings represent critical factors that will form the foundation of future research endeavors aimed at enhancing plant regeneration strategies, thereby advancing various cultivation and biotechnological applications related to this economically significant crop. The absence of a robust embryogenic system capable of yielding vigorous plants directly from cotyledon embryos represents a substantial challenge in the pursuit of understanding callus development on a deeper level. In our study, the term “embryogenic” refers to the observed developmental trajectory and molecular signatures of calli that exhibit specific characteristics suggestive of embryogenic potential. However, without the ability to produce fully developed, viable plants directly from these calli, we must exercise caution when labeling them as traditional embryogenic calli, that is, callus capable of producing somatic embryos. This work marks an essential initial step in unraveling the intricacies of cannabis callus development. Although we have identified notable distinctions in gene expression profiles characteristic of specific calli types and have highlighted key regulatory factors, it remains crucial to be cognizant of the possibility that these so-called “embryogenic” tissues may follow an unfamiliar developmental path. Such a path could be distinct from the conventional embryogenic pathway that leads to plant regeneration. To address this limitation and ensure the validity of our findings, future research endeavors should aim to establish a more robust and reproducible embryogenic system in cannabis. This would enable a direct comparison between calli capable of developing into healthy, vigorous plants and those that are not. Ultimately, future research in this area will allow us to better understand the molecular mechanisms underpinning successful plant regeneration. Conducting additional experiments to validate the functional roles of the identified genes is essential and may be achieved by incorporating CRISPR-based functional validation methods to more comprehensively determine the molecular mechanisms driving cannabis callus development.

## Figures and Tables

**Table 1:** A list of the top 10 upregulated and downregulated genes in embryogenic calli.

Gene ID	Gene Description	Base Mean	log_2_FC	Stat	*p*-Value	Padj	RC	NEC	EC
Upregulated genes									
LOC115706125	beta-galactosidase	1935.192	4.532471	21.99906	2.94E-107	1.61E-103	2271.333	123.3333	4521.667
LOC115725246	receptor-like protein 35	544.5763	4.741848	20.13178	3.89E-90	1.28E-86	1027.333	21	917.3333
LOC115707713	programmed cell death protein 4-like	1672.396	4.035308	18.47184	3.48E-76	9.52E-73	2525	127.6667	3351.667
LOC115723447	1-aminocyclopropane-1-carboxylate oxidase homolog 1	1043.549	3.383501	16.56611	1.23E-61	1.83E-58	1507.333	124.6667	2067.667
LOC115709330	guanine nucleotide-binding protein subunit beta-like protein	1077.133	2.700104	15.26122	1.39E-52	1.14E-49	1922.333	165.6667	1726.667
LOC115705178	60S ribosomal protein L31	666.7065	7.191924	16.19705	5.29E-59	6.68E-56	1300.667	4.666667	1128
LOC115705507	F-box/LRR protein	394.5115	2.516052	15.25394	1.55E-52	1.21E-49	627	74	686
LOC115705442	acid phosphatase 1	462.3999	3.911658	14.80861	1.29E-49	9.61E-47	721.6667	37.66667	896.3333
LOC115714995	protein DETOXIFICATION 29	391.0912	5.559223	15.69295	1.69E-55	1.73E-52	671.6667	9.666667	724.6667
LOC115705054	60S acidic ribosomal protein P1	561.4693	7.425328	15.71247	1.24E-55	1.36E-52	1021.667	3.666667	1013.333
Downregulated genes									
LOC115705878	beta-galactosidase-like	2081.346	−10.283	−25.6555	3.7E-145	6E-141	7.573207	6231.446	5.020056
LOC115704132	LRR receptor-like serine/threonine-protein kinase IOS1	578.948	−4.85181	−22.9363	2E-116	1.7E-112	73.00982	1608.248	55.58613
LOC115716639	metal-nicotianamine transporter YSL3	412.6694	−4.14058	−21.2908	1.4E-100	5.67E-97	130.2399	1048.471	59.29757
LOC115706817	protein TIFY 10A-like	273.1394	−6.44788	−17.0166	6.18E-65	1.13E-61	12.01685	798.2773	9.124129
LOC115711284	SNF1-related protein kinase regulatory subunit gamma-1	358.2644	−4.13545	−16.7441	6.25E-63	1.03E-59	51.28097	968.5667	54.94563
LOC115721742	peroxidase 24	166.997	−4.47462	−16.4257	1.25E-60	1.71E-57	10.53092	469.3534	21.10658
LOC115702721	probable 2-oxoglutarate-dependent dioxygenase At3g50210	446.0996	−5.51294	−16.1715	8.01E-59	9.4E-56	15.63127	1294.354	28.31303
LOC115702931	ferredoxin--nitrite reductase, chloroplastic-like	425.0991	−4.18426	−15.4685	5.66E-54	5.16E-51	98.75676	1115.158	61.38266
LOC115697518	neutral ceramidase 2	119.8976	−5.47992	−14.648	1.39E-48	9.48E-46	18.49675	333.8255	7.37046
LOC115709323	guanine nucleotide-binding protein subunit beta-like protein	451.4577	−2.67595	−12.5052	6.99E-36	2.5E-33	160.609	1032.158	161.6061

RC: rooty callus; NEC: non-embryogenic callus; EC: embryonic callus.

## Data Availability

All data generated or analyzed during this study are included in this published article as supplementary information files.

## Supplementary Materials

The following supporting information can be downloaded at: https://www.mdpi.com/article/10.3390/ijms241914625/s1.
